# Assessing data availability for the development of REDD-plus national reference levels

**DOI:** 10.1186/1750-0680-5-6

**Published:** 2010-09-30

**Authors:** Chisa Umemiya, Masahiro Amano, Suphawadee Wilamart

**Affiliations:** 1 School of Human Sciences, Waseda University, Mikajima 2-579-15, Tokorozawa, Saitama, 359-1192, Japan; 2 Japan Society for the Promotion of Science, Chiyoda-ku 1-8, Tokyo, 102-8472, Japan; 3 Permission Division, Royal Forest Department, 61 Phahonyothin Rd., Ladyao, Chatuchak, Bangkok 10900, Thailand

## Abstract

**Background:**

Data availability in developing countries is known to be extremely varied and is one of the constraints for setting the national reference levels (RLs) for the REDD-plus (i.e. 'Policy approaches and positive incentives on issues relating to reducing emissions from deforestation and forest degradation in developing countries; and the role of conservation, sustainable management of forests and enhancement of forest carbon stocks in developing countries') under the UNFCCC. Taking Thailand as a case study country, this paper compares three types of RLs, which require different levels of datasets, including a simple historic RL, a projected forest-trend RL, and a business-as-usual (BAU) RL.

**Results:**

Other than the finding that different RLs yielded different estimations on future deforestation areas, the analysis also identified the characteristics of each RL. The historical RL demanded simple data, but can be varied in accordance with a reference year or period. The forest-trend RL can be more reliable than the historical RL, if the country's deforestation trend curve is formed smoothly. The complicated BAU RL is useful as it can demonstrate the additionality of REDD-plus activities and distinguish the country's unintentional efforts.

**Conclusions:**

With the REDD-plus that involves widespread participation, there should be steps from which countries choose the appropriate RL; ranging from simpler to more complex measures, in accordance with data availability in each country. Once registered with REDD-plus, the countries with weak capacity and capability should be supported to enhance the data collection system in that country.

## Background

Around 13 million hectares of global forest area is being lost every year largely in the tropics [1]. It is estimated that, following the burning of fossil fuels, tropical deforestation is the second largest source of greenhouse gas emissions [2]. Recognising the critical role of tropical forests in combating climate change, it is expected that an agreement under the UN Framework Convention on Climate Change (UNFCCC) will be made with regard to: 'Policy approaches and positive incentives on issues relating to reducing emissions from deforestation and forest degradation in developing countries; and the role of conservation, sustainable management of forests and enhancement of forest carbon stocks in developing countries' ('REDD-plus') [3,4]. REDD-plus could contribute to the mitigation of climate change only if various methodological issues are resolved, including how to set country-specific reference levels (RLs). National RLs are used to determine the level below which the countries' reduced emissions could be measured and credited. Desirable RLs must ensure that rewarded emissions are additional; while they should also encourage widespread participation, as they are directly linked with REDD-plus incentives.

**Figure 1:**
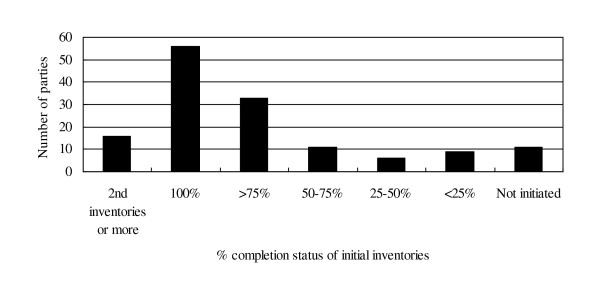
**The completion status of GHG inventories in NCs by non-Annex I parties**.

To date, there is convergence that RLs should be based on historic data, while taking into account national circumstances [5]. However, it is yet to be clear how REDD-plus countries should establish the RLs in practical terms. While a number of proposals have been made by parties and observers, one of the important factors that countries would need to consider when selecting the RLs is the availability of data [6-12]. We review in Figure 1 the completion status of greenhouse gas (GHG) inventories, as part of national communications (NC), by developing (non-Annex I) parties to UNFCCC [13]. The land use change sector is one component of the inventories, and should be estimated based on the guidance and guidelines of the Intergovernmental Panel on Climate Change (IPCC). Currently, there is convergence that the most recent IPCC guidance and guidelines should also be used for REDD-plus [5]. Thus, it is useful to compare different levels of experience in the preparation of inventories, which could indicate some disparities in countries' capacities for estimating RLs, including the availability of data. Among 142 non-Annex I parties, half of them have completed the inventories at least once; while others are still in the process of doing so, of which some are still to initiate work. Of those currently preparing for the submission of the first inventories, a difference in progress is evident. Therefore, it is highly likely that some participating developing countries may have limited data to establish RLs in comparison to others. In other words, without fully taking into account such disparities in data availability among countries, there would be difficulty in ensuring widespread participation in REDD-plus [8].

This paper presents three types of RLs, which require different data levels, by taking Thailand as a case study country. We then evaluate the applicability of each under REDD-plus. Thailand was suitable for this study for two major reasons: 1) it had sufficient types of data to develop three different RLs, including those relevant to national policy approaches to prevent deforestation, which were the commercial logging ban in natural forests in 1989 and the protected area system (i.e., national parks and wildlife sanctuaries) starting from 1961; and 2) those data were available over a long period of time (e.g., from 1975 to 2003).

## Methods

In this study, we compare three types of RLs, which require different levels of data (Table 1) [14-19]. The data were available only for estimating RLs on deforestation in terms of forest area, defined as *'forest of all types ... with an area of 5 hectares or more with trees taller than 5 metres or more and with canopy covering more than 10% of the ground area' *[18]. The first and simplest RL is a historical RL; based on the annual deforestation area in the past period [20]. We estimated the annual deforestation area in a year and averaged that over five years to smooth the yearly variation. The second RL is referred to as a forest-trend RL, employing a quadric curve to the time-series forest area data. With the estimated deforestation trend curve, the future forest area was predicted. The third and most complex RL of this study is a business-as-usual (BAU) RL, which can project the future deforestation by incorporating a large number of variables, including key socioeconomic, technological, and political factors that drive deforestation [6]. We used the econometric model to estimate a BAU RL, as it can be used to simulate the link between the chosen variables and deforestation [10,21,22]. The econometric model of our analysis consisted of two parts: one representing annual changes in area of three land use classes (i.e., forest, farmland, and unclassified land); and the other reflecting the variables linked to those changes. Because a BAU RL represents the relationship between the variables and deforestation, it was possible to predict future deforestation under the hypothesised scenarios, under which we modified the values of the variables. Three scenarios were taken with the BAU RL, including: a Standard scenario; a Conservation scenario; and an Industrialisation scenario (see Table 2). In the Standard scenario, we assumed that the variables would change in a constant manner in the period between 2000 and 2003. In the Conservation scenario, we hypothesised that the country's protected area, which includes national parks and wildlife sanctuaries, would be doubled compared with the period between 2000 and 2003, while other variables would be the same as the Standard scenario. The industrialisation scenario differs from the Standard scenario as it assumed that two industry-related variables, productivity for major agricultural crops and GDP in the non-agriculture sectors, would be increased at an accelerated rate than that which happened between 2000 and 2003.

**Table 1:** Variables used in the analysis

RL	Variable	Unit	Source
Historical	Forest area	10^3 ^ha	[14]
Forest-trend	Forest area	10^3 ^ha	[14]
BAU	Land use area (forest, farmland, unclassified land)	10^3 ^ha	[14]
	Population	10^3 ^persons	[15]
	GDP at the country level	10^9 ^Baht	[16]
	Sectoral GDP (in agriculture, manufacture, construction, sales, transportation, finance, public)	10^9 ^Baht	[16]
	Production of major agricultural crops	10^3 ^tonnes	[17]
	Production of major meat	10^3 ^tonnes	[17]
	Production of wood products	10^3 ^m^2^	[18]
	Productivity for major agricultural crops*		
	Area of national parks and wildlife sanctuaries	10^3 ^ha	[19]

*'Production of major agricultural crops' divided by area of 'farmland'.

**Table 2:** Scenario design for a BAU RL

Scenario option	Modification on variables
Standard	All the variables adjusted at the changing rate equal to 2000-2003.
Conservation	Annual increased area of national parks and wildlife sanctuaries adjusted to be 100,000 ha from 50,000 between 2000 and 2003; and other variables consistent with the Standard.
Industrialisation	Annual growth rate of productivity for major agricultural crops and GDP in non-agriculture sector (i.e., manufacture, construction, sales, transportation, and finance) adjusted to be 10% from 7% and 5% in 2000-2003, respectively; and other variables consistent with the Standard.

Depending on the period for which the necessary data were available, the historical and forest-trend RLs were developed for 1975 to 2003, and the BAU baseline for 1981 to 2003. Then taking a year, 2013, as an example, which is right after the Kyoto Protocol's first commitment period, we compared the values of the forest area by the three RLs.

It was reported that there was discrepancy of the data on the forest area before and after 2000, since the data after 2000 lacks ground truth surveys and deviates significantly from earlier figures [23]. Therefore, we estimated values on the forest area after 2000 by a liner extrapolation of the last values between 1995 and 1999. The differences between the original and calibrated values on the forest area were added to unclassified land. To reflect this adjustment on land use data, we used a dummy variable in the econometric model when developing a BAU RL.

## Results

### Historical RL

**Figure 2:**
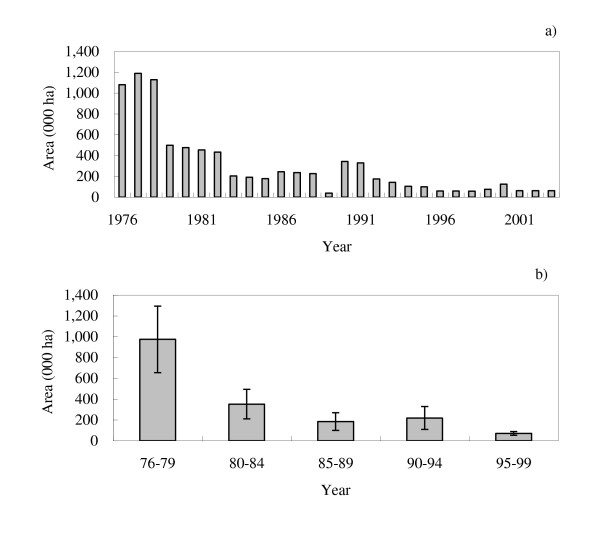
**Historical RLs: annual deforestation area (a) and annual deforestation area averaged for five years (b)**. Vertical bars in (b) indicate the standard deviation of values.

The area of forest loss per year and that averaged for the period of five years are presented in Figure 2(a) and 2(b), respectively. The annual loss of forest area is considerably larger in earlier rather than recent years, regardless of the values being averaged over some years or not. If we take reference years of 1985 and 1995, for example, the estimated values were 178,642 ha and 99,743, respectively. Instead, if we follow a 5-year reference period of between 1985 and 1989 and 1995 and 1999, the figures were estimated as 184,711 ha and 70,122, respectively.

### Forest-trend RL

The quadric model based on the historical trend of the forest area is as follows:

FOR = 12.355t^2 ^- 49388t + 4.937*10^7^

(adj. R^2^: 0.965; S.E.: 416.509)

Where: *FOR*, forest area; *t*, year

**Figure 3:**
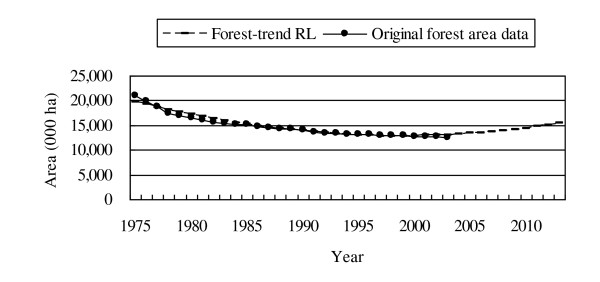
**Comparison of the forest-trend RL and the original data on forest area**.

The fitness of the model appears high (adjusted R^2 ^= 0.965; see Figure 3), which means that the projected trend of deforestation is likely. However, this is possible only under the condition of a country, such as Thailand, where the deforestation trend shapes a relatively smooth curve.

### BAU RL

**Figure 4:**
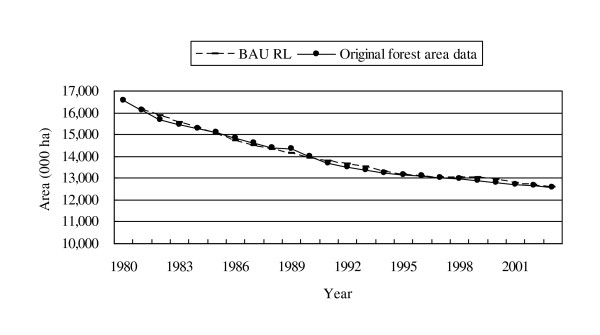
**Comparison of the BAU RL and the original data on forest area**.

The components of the econometric model to estimate a BAU RL are presented in Table 3. The results of the model showed a satisfactorily high projection level (Figure 4). In the model, it is interpreted that as agricultural GDP increased and productivity for major agricultural crops and implementation of the protected area system decreased, the area of new farmland was likely to have expanded. This means that as more economic demand was placed on the agriculture industry, the area of farmland was assumed to have increased, while the improvement of productivity for major agricultural crops and the protected area system was likely to have reduced such pressure on new farmland. Unclassified land, on the other hand, was likely to have enlarged, as GDP in the non-agriculture sector increased. This indicates that as the country has become more industrialised and urbanised, more land was categorized as unclassified land, which includes, such areas as cities, industrial sites, and various types of infrastructure.

**Table 3:** The econometric model for a BAU RL

${\text{FOR}}_{\left(\text{t}\right)}=\underset{\left(1.864\right)}{21274.88}+\underset{\left(2.562\right)}{0.575}*{\text{FOR}}_{\left(\text{t}-1\right)}\underset{\left(-8.655\right)}{-0.918}*{\text{FA}}_{\left(\text{t}\right)}+\underset{\left(2.036\right)}{0.501}*{\text{FA}}_{\left(\text{t}-1\right)}\underset{\left(-9.895\right)}{-0.877}*{\text{UNC}}_{\left(\text{t}\right)}+\underset{\left(1.808\right)}{0.439}*{\text{UNC}}_{\left(\text{t}-1\right)}\underset{\left(-0.541\right)}{-18.514}*{\text{D}}_{\left(\text{t}\right)}$ *(adj. R^2^: 0.999; S.E.: 31.956)*
${\text{FA}}_{\left(\text{t}\right)}=\underset{\left(3.956\right)}{3745.355}+\underset{\left(15.248\right)}{0.816}*{\text{FA}}_{\left(\text{t}-1\right)}\underset{\left(-2.216\right)}{-22.064}*{\text{PDT}}_{\left(\text{t}\right)}+\underset{\left(1.843\right)}{5.315}*{\text{GDPA}}_{\left(\text{t}\right)}\underset{\left(-0.888\right)}{-0.0460}*{\text{NPWS}}_{\left(\text{t}\right)}$ *(adj. R^2^: 0.950; S.E.: 109.500)*
${\text{UNC}}_{\left(\text{t}\right)}=\underset{\left(2.405\right)}{4009.106}+\underset{\left(6.646\right)}{0.734}*{\text{UNC}}_{\left(\text{t}-1\right)}+\underset{\left(2.820\right)}{0.325}*{\text{GDPN}}_{\left(\text{t}\right)}+\underset{\left(0.803\right)}{79.238}*{\text{D}}_{\left(\text{t}\right)}$ *(adj. R^2^: 0.972; S.E.: 131.306)*

Where: *FOR*, forest area; *t*, year; *FA*, area of farmland; *UNC*, area of unclassified land; *D*, a dummy variable; *PDT*, productivity for major agricultural crops; *GDPA*, GDP in agricultural sector; *NPWS*, area of national parks and wildlife sanctuaries; *GDPN*, GDP in non-agricultural sector

**Figure 5:**
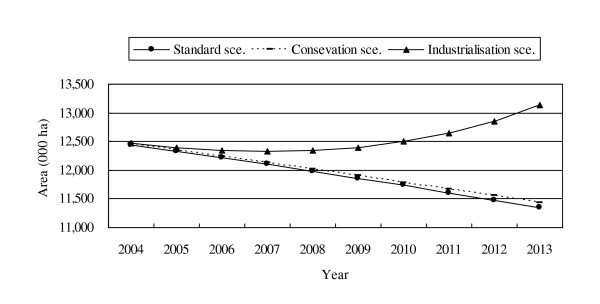
**Projections for forest area by the BAU RL with three scenarios**.

With the model, BAU RLs with three different scenarios, as explained before, were estimated for the years after 2003 as shown in Figure 5. Among the three BAU RLs, deforestation continued under the Standard and Conservation scenarios; while in the Industrialisation scenario, the forest area started to recover then increase. The incremental growth of forest area under the Industrialisation scenario could be caused by factors such as the natural regeneration of forests, where there once was farmland, and increased tree plantations. The deforested area in the Conservation scenario is slightly less than that in the Standard, which obviously is associated with the enhanced protected area system under assumption.

### Comparison of the three RLs

Projection on the forest area for 2013 as a sample national RL is summarised (Table 4). To compare the findings easily with the other two RLs, the results of the historical RL were converted from the annual deforestation area to the forest area, based on the value of the forest area in 2003 (i.e., the estimated annual deforestation area in 2013 was deducted from the 2003 figure for forest area). The forest-trend RL and BAU RL for the Industrialisation scenario produced a high estimation of the forest area, which was above the forest area in 2003. The results of the other RLs, especially of the BAU RL for the Standard and Conservation scenarios, appeared low compared with the situation in 2003.

**Table 4:** Projected forest area for 2013 by three RLs

RL		Area (000 ha)	% ratio to forest area in 2003
Historical			
	*With sample base yr/period of:*		
Yearly	1985	12,405	98.6%
	1995	12,484	99.2%
Periodical	1985-89	12,399	98.5%
	1995-99	12,513	99.4%
Forest trend		15,318	121.7%
BAU			
Standard sce.		11,351	90.2%
Conservation sce.		11,427	90.8%
Industrialisation sce.		13,130	104.3%

## Discussion

The characteristics of each of the three RLs, which required different levels of data, can be highlighted. First of all, not surprisingly, different RLs yielded different estimations for the future forest area. Our analysis showed that a RL with limited data (i.e., historical and forest-trend RLs) did produce a higher estimation of forest area than RLs with a large number of datasets (i.e., a BAU RL with the Standard and Conservation scenarios). This reminds us that the data availability of a country can influence not only the choice of a national RL, but also the REDD-plus incentives that it can receive. In this context, the quality of data used to develop a RL is also critical, thus should be reported and evaluated at the national registration with REDD-plus by, for instance, following the IPCC guidance and guidelines.

Secondly, it was found that the simple historical RL could be varied considerably, depending on the selection of a base year or period; while the forest-trend RL, which is relatively simple and likely to be more reliable than the historical RL, could be useful, only when the country's deforestation curve is formed smoothly. We recommend that these types of RLs based on a smaller number of datasets should be selected for countries which have less capacity and capability, including collection of data. We believe that a relaxation of requirements for a national RL, especially for the countries with limited data, is essential to realise widespread participation in REDD-plus. As an international treatment, REDD-plus has to be accessible to all developing parties. Besides, involving as many countries as possible is important for the carbon effectiveness of REDD-plus, because it can minimise a risk for the international leakage of forest emissions (i.e., reduced emissions occurring outside REDD-plus participating countries). Therefore, we suggest that the developing countries which have less capacity and capability should be able to adopt the RL that their best available data permits. Nevertheless, to avoid possible unfavorable 'hot air' (i.e., credited emissions without a country's additional efforts) caused by adopting a simpler type of RLs, the countries are recommended to develop RLs in a carbon conservative manner when registering with REDD-plus. Further, we propose that once registered with REDD-plus, the participating countries, if necessary, should be able to enhance their capacity and capability, which could include, as appropriate, an improved data collection system. With that improved data condition, they may be able to apply a more sophisticated RL, based on a large number of data types, such as a BAU RL, as introduced in this study.

Thirdly, other than the fact that it reflects the national circumstances related to deforestation, the BAU RL with a wide range of data types showed its strengths which can not be seen with the other simpler RLs (i.e., historical and forest-trend RLs). First, it could demonstrate the effects of the country's policy approaches to reduce deforestation. This can be useful for identifying the country's efforts that are additional because of REDD-plus. Such information should be valuable not only for national policy makers addressing deforestation, but also at the registration process of national REDD-plus action plans at the UNFCCC level. Second, the BAU RL could eliminate the unintended effects of a country's development on reduced deforestation. The analysis of this study showed that industrialisation in Thailand could help to reduce national deforestation. Nonetheless, industrialisation in many parts of the developing world (e.g., in China, India, and other fast growing countries), is likely to be promoted even without REDD-plus incentives. Therefore, it is necessary to prevent such unintentional effects from being counted as part of REDD-plus credits by using a detailed RL, such as the BAU RL of this study.

## Conclusions

Given that data availability in developing countries is extremely varied, we suggest that countries participating in REDD-plus should be able to use the RL that fits into the situation which relates to their available datasets. To do this, REDD-plus must prepare the steps from which countries can choose the appropriate RL; ranging from simpler to more complex measures in accordance with their data availability. Support from developed countries to the once registered REDD-plus countries must be in place, so that countries with insufficient capacity and capability could strengthen the data collection system in that country, which can be used later to establish a more sophisticated RL. The REDD-plus that gains wide participation can contribute to not only the mitigation of climate change, but also the opportunity to manage tropical forests in a sustainable way, which could also benefit a number of other forest functions and services, including such as biodiversity, water catchment, prevention of soil runoff, and stabilisation of local climate.

## Competing interests

The authors declare that they have no competing interests.

## Authors' contributions

CU carried out the study, performed the statistical analysis, and drafted the manuscript. MA participated in the design of the study and the development of the models. SW developed the database and acquired secondary information. All authors improved and approved the final manuscript.
